# Association between sense of coherence and health and well-being among older survivors of a natural disaster: a prospective outcome-wide study

**DOI:** 10.1038/s41598-023-43672-z

**Published:** 2023-09-29

**Authors:** Hiroyuki Hikichi, Koichiro Shiba, Jun Aida, Katsunori Kondo, Ichiro Kawachi

**Affiliations:** 1 Kitasato University School of Medicine, 1-15-1 Kitazato, Minami, Sagamihara, Kanagawa 252-0374 Japan; 2 Department of Epidemiology, Boston University School of Public Health, 715 Albany Street, Boston, MA 02118 USA; 3 Department of Oral Health Promotion, Graduate School of Medical and Dental Sciences, Tokyo Medical and Dental University, 1-5-45 Yushima, Bunkyo, Tokyo, 113-8510 Japan; 4 Center for Preventive Medical Sciences, Chiba University, 1-8-1 Inohana, Chuo, Chiba, Chiba 260-8670 Japan; 5 Center for Gerontology and Social Science, National Center for Geriatrics and Gerontology, 7-430 Morioka, Obu, Aichi 474-8511 Japan; 6 Department of Social and Behavioral Sciences, Harvard T.H. Chan School of Public Health, 677 Huntington Avenue, Boston, MA 02115 USA

**Keywords:** Human behaviour, Risk factors

## Abstract

We examined whether pre-disaster Sense of Coherence (SOC) mitigated the impact of housing damage on health and well-being of older survivors after the 2011 Japan Earthquake and Tsunami. A panel survey was conducted in a city located 80 km west of the epicenter seven months before and three years after the disaster (3594 respondents). Among respondents with lighter property damage, higher SOC was inversely associated with mental distress (coefficient − 0.29, 95% CI (confidence interval) − 0.39, − 0.19, *p* < .01), unhappiness (coefficient − 0.33, 95% CI − 0.43, − 0.23, *p* < .01), low expectation of mutual help (coefficient − 0.17, 95% CI − 0.27, − 0.07, *p* < .01), and weak community attachment (coefficient − 0.20, 95% CI − 0.30, − 0.11, *p* < .01). Conversely, among those who experienced housing loss, higher SOC was no longer protectively associated with health and well-being. Loss of generalized resistance resources due to serious damage led to difficulties in stress coping.

## Introduction

Older individuals represent a particularly vulnerable population in the aftermath of natural disasters. Research has demonstrated that disaster experiences can adversely affect multiple domains of health and well-being, including risks of posttraumatic stress disorder^1^, depression^2^, physical inactivity^3^, obesity^4^, cardiovascular diseases^5^, functional limitations^6^, cognitive decline^7^, and social isolation^8^.

The concept of sense of coherence (SOC) was originally formulated by Aaron Antonovsky (1979) to explain why some people become sick under stress while others stay healthy^9^. According to Antonovsky, SOC is a global psychological orientation expressed by a set of beliefs about the world: (1) comprehensibility—that every life event happening to oneself is understandable and can be foreseen because the world is ordered and structured; (2) manageability—that one possesses the competence to cope effectively with problems in life; and (3) meaningfulness— that solving difficult problems is worthwhile and reasonable^10^. People who have high SOC are therefore more resilient and better equipped to handle psychological stressors, resulting in protective effects on health^11^ and well-being^12^_._ A cross-sectional study of Swedish adult survivors of the 1994 Estonia ferry disaster (n = 42) suggested an inverse association between higher levels of SOC and greater Posttraumatic Stress (PTSS) severity^13^. Another study investigated radiation-related anxiety among Japanese public health nurses (n = 458) after the Fukushima Nuclear Power Station accident, showing a cross-sectional association between higher scores of SOC and lower levels of anxiety^14^.

The previous studies have two major limitations. Most of the studies measured SOC after traumatic events, which are subject to reporting bias, survivorship bias, and reverse causation. They also have not considered pre-exposure outcomes in analyses, which might have increased the probability of selection bias. Therefore, research has yielded inconsistent findings regarding the association of SOC and health among trauma survivors.

In this study, we examined the hypothesis that sense of coherence is associated with better health and well-being even among victims of a massive natural disaster. To comprehensively capture the range of health outcomes potentially associated with SOC, we used a longitudinal outcome-wide approach to assess whether SOC before a disaster buffers the impact of disaster experiences on the health and well-being of older survivors of the 2011 Japan earthquake and tsunami. This study mainly focused on housing damage as a primary indicator of disaster experiences because our previous outcome-wide study demonstrated that housing damage is a unique predictor for various types of health outcomes and well-being^15^.

**Figure 1:**
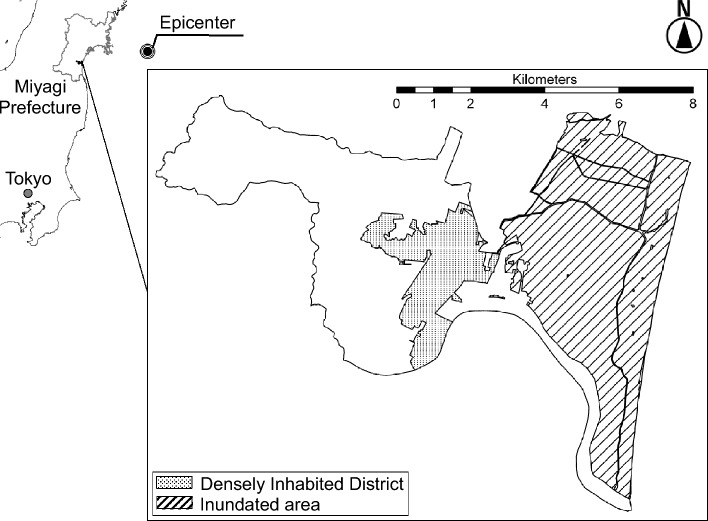
Map of tsunami-inundated area in Iwanuma City, Japan.

Study subjects were drawn from a nationwide cohort study of aging, called the Japan Gerontological Evaluation Study (JAGES), which was established seven months before the disaster. One of the field sites of the cohort was Iwanuma City in Miyagi Prefecture, located approximately 80 km west of the earthquake epicenter (Fig. 1). The pre-disaster baseline survey inquired about sense of coherence, mental and physical health, health behaviors, and patterns of social participation, social support, pro-social attitudes, and other factors. Approximately 2.5 years after the disaster, we managed to locate 82.1% of the survivors to gather information about their disaster experiences and the same set of variables regarding health outcomes and social connectedness. In addition, we linked their panel data to the incidence of physical and cognitive impairment ascertained by in-home assessment and medical examination under Japan’s national Long-Term Care Insurance (LTCI) registry. This unique design affords us to use SOC and a comprehensive set of outcomes before the disaster.

## Results

Comparing our analytic sample with data from the local census at the baseline (Table S1), we can see that the sex distribution in our analytic sample (55.4% women) is quite comparable to the actual census of older residents conducted in the city of Iwanuma in October 2010 (female 57.2%)^16^. The age distribution of our sample is younger than the local census data (respondents 62.1% vs. census data 51.8%, for groups aged 65–74 years)^16^. A higher proportion of respondents in our sample were married (74.8%) compared to the census data (64.7%)^16^. The proportion of working people in our data (18.6%) is also close to the census data (17.2%)^16^. These comparisons support the representativeness of our data with respect to the entire older population of Iwanuma.

We implemented a confirmatory factor analysis to check the construct validity of sense of coherence measured by six questions. As shown in Figure S1, we found a reasonable three-factor solution with acceptable goodness of fit indices (comparative fit index (CFI) 0.998, root-mean-square error of approximation (RMSEA) 0.023, standardized-root-mean-square residual (SRMA) 0.011). We calculated the arithmetical mean of the six items to create the total SOC score (mean 3.69, standard deviation (SD) 0.70) because of high correlations among the three factors, which indicated good internal consistency (Cronbach’s α = 0.83). And then, we created a binary variable that categorized participants into two groups using a median split (median 3.67).

Table 1 presents demographic characteristics at the baseline, disaster experiences, and each outcome at the follow-up survey by levels of SOC. There were no remarkable differences in baseline characteristics between the lower and higher SOC groups except for equivalized household income (216.09 JPY vs. 249.55 JPY). The proportions of respondents who experienced housing destruction and loss of loved ones were close between the two groups (5.0% vs. 3.0%, and 39.0% vs. 36.1%, respectively). However, respondents with higher SOC exhibited better health and well-being in the follow-up survey in comparison to those with lower SOC aside from current smoking and drinking alcohol (7.9% vs. 8.6%, and 31.9% vs. 35.5%, respectively).

**Table 1:** Characteristics of the analytic samples.

	Overall sample	Lower SOC (n = 1515)^a^	Higher SOC (n = 1528)^a^
	n (%)/mean (SD)	n (%)/mean (SD)	n (%)/mean (SD)
Outcomes at the follow-up survey			
Cognitive health			
Levels of cognitive decline assessed by medical doctors, mean (SD)	1.13 (0.63)	1.15 (0.68)	1.08 (0.52)
Levels of cognitive decline assessed by trained investigators, mean (SD)	1.16 (0.66)	1.18 (0.71)	1.10 (0.51)
Physical health			
Levels of physical disability, mean (SD)	1.30 (1.04)	1.35 (1.12)	1.20 (0.88)
Impaired higher-level IADL (TMIG-IC), mean (SD)	1.73 (2.57)	1.99 (2.74)	1.24 (2.11)
Missing, n (%)	178 (5.31)	66 (4.4)	70 (4.6)
Less remained teeth, mean (SD)	2.13 (1.36)	2.20 (1.39)	1.97 (1.28)
Missing, n (%)	82 (2.45)	39 (2.6)	15 (1.0)
Incident of fall, n (%)	193 (5.8)	112 (7.4)	57 (3.7)
Missing, n (%)	22 (0.7)	9 (0.6)	7 (0.5)
Obesity, n (%)	913 (27.3)	427 (28.2)	408 (26.7)
Missing, n (%)	96 (2.9)	39 (2.6)	37 (2.4)
The number of existing diseases, mean (SD)	1.70 (1.33)	1.80 (1.38)	1.58 (1.27)
Mental health			
Mental distress (K6), mean (SD)	4.19 (4.20)	5.63 (4.40)	2.70 (3.34)
Missing, n (%)	133 (4.0)	74 (4.9)	35 (2.3)
Depressive symptoms (> = 5 points of GDS-15 score), n (%)	911 (27.2)	602 (39.7)	213 (13.9)
Missing, n (%)	427 (12.8)	196 (12.9)	148 (9.7)
Posttraumatic stress symptoms (> = 6 points of SQD score), n (%)	357 (10.7)	226 (14.9)	90 (5.9)
Missing, n (%)	196 (5.9)	92 (6.1)	67 (4.4)
Poor sleep quality, mean (SD)	2.21 (0.65)	2.34 (0.67)	2.08 (0.60)
Missing, n (%)	52 (1.6)	26 (1.7)	17 (1.1)
Health behavior			
Less daily walking time, mean (SD)	2.90 (1.03)	2.94 (1.02)	2.81 (1.04)
Missing, n (%)	41 (1.2)	17 (1.1)	15 (1.0)
Decreased frequency of going out in the past year, n (%)	835 (24.9)	441 (29.1)	282 (18.5)
Missing, n (%)	89 (2.7)	37 (2.4)	34 (2.2)
Current smoking, n (%)	271 (8.1)	119 (7.9)	132 (8.6)
Missing, n (%)	21 (0.6)	8 (0.5)	9 (0.6)
Current drinking, n (%)	1097 (32.8)	483 (31.9)	542 (35.5)
Missing, n (%)	23 (0.7)	10 (0.7)	9 (0.6)
Subjective well-being			
Low self-rated health, mean (SD)	2.14 (0.64)	2.25 (0.65)	2.01 (0.59)
Missing, n (%)	26 (0.8)	16 (1.1)	7 (0.5)
Subjective unhappiness, mean (SD)	3,87 (1.85)	4.45 (1.83)	3.26 (1.60)
Missing, n (%)	85 (2.5)	40 (2.6)	27 (1.8)
Social well-being			
Low likelihood of mutual help in the local community, mean (SD)	2.45 (0.79)	2.57 (0.83)	2.34 (0.73)
Missing, n (%)	73 (2.2)	37 (2.4)	20 (1.3)
Less trust to local residents, mean (SD)	2.25 (0.71)	2.34 (0.74)	2.15 (0.64)
Missing, n (%)	47 (1.4)	17 (1.1)	10 (0.7)
Weak community attachment, mean (SD)	2.04 (0.82)	2.20 (0.86)	1.87 (0.72)
Missing, n (%)	43 (1.3)	21 (1.4)	8 (0.5)
Low frequency of meeting friends, mean (SD)	3.32 (1.55)	3.47 (1.52)	3.19 (1.53)
Missing, n (%)	45 (1.3)	18 (1.2)	16 (1.0)
Low frequency of participating in sports clubs, mean (SD)	5.18 (1.44)	5.30 (1.35)	4.99 (1.55)
Missing, n (%)	135 (4.0)	50 (3.3)	70 (4.6)
Low frequency of participating in hobby clubs, mean (SD)	5.06 (1.40)	5.17 (1.32)	4.88 (1.47)
Missing, n (%)	130 (3.9)	52 (3.4)	62 (4.1)
Small number of friends meeting in the past month, mean (SD)	3.47 (1.34)	3.30 (1.32)	3.68 (1.33)
Missing, n (%)	52 (1.6)	26 (1.7)	13 (0.9)
Unwillingness to cooperate with the local community, mean (SD)	1.64 (0.57)	1.72 (0.57)	1.54 (0.57)
Missing, n (%)	129 (3.9)	62 (4.1)	40 (2.6)
Few emotional social support, mean (SD)	4.00 (1.28)	4.07 (1.24)	3.85 (1.31)
Few instrumental social support, mean (SD)	4.36 (0.94)	4.42 (1.24)	4.25 (0.95)
Disaster experiences			
Housing damage			
None, n (%)	1334 (39.8)	565 (37.3)	654 (42.8)
Milder damage, n (%)	1778 (53.1)	827 (54.6)	801 (52.4)
Housing destruction, n (%)	148 (4.4)	76 (5.0)	46 (3.0)
Missing, n (%)	90 (2.7)	47 (3.1)	27 (1.8)
Loss of loved ones, n (%)	1254 (37.4)	591 (39.0)	552 (36.1)
Baseline characteristics			
Age, mean (SD)	73.20 (5.99)	72.9 (5.83)	72.9 (6.00)
Female, n (%)	1857 (55.4)	829 (54.7)	805 (52.7)
Education (1: < 6 years–4: ≧ 13 years), mean (SD)	2.86 (0.78)	2.78 (0.75)	2.98 (0.78)
Missing, n (%)	95 (2.8)	18 (1.2)	10 (0.7)
Equivalized household income (10,000 JPY), mean (SD)	230.63 (141.11)	216.09 (140.77)	249.55 (140.33)
Missing, n (%)	388 (11.6)	245 (16.2)	196 (12.8)
Working, n (%)	550 (18.6)	230 (15.2)	285 (18.7)
Missing, n (%)	388 (11.6)	151 (10.0)	117 (7.7)
Divorce or bereavement, n (%)	816 (24.4)	380 (25.1)	356 (23.3)
Missing, n (%)	170 (5.1)	52 (3.4)	40 (2.6)
Living alone, n (%)	281 (8.4)	143 (9.4)	111 (7.3)
Missing, n (%)	90 (2.7)	15 (1.0)	8 (0.5)
Higher Sense of coherence, n (%)	1528 (45.6)	–	–
Missing, n (%)	307 (9.2)	–	–

JPY, Japanese Yen; GDS-15, Geriatric Depression Scale-15; TMIG-IC, Tokyo Metropolitan Institute of Gerontology Index of Competence; SQD, Screening Questionnaire for Disaster mental health.

^a^The total number of participants in lower and higher SOC groups is not same as the analytic sample (n = 3350), because of missing answers on SOC measurements.

Table S2 shows the prevalence of baseline outcomes by levels of housing damage. In comparison with respondents with no housing damage and milder housing damage, those with housing loss had more impaired higher-level IADL (1.07, 1.11, and 1.58, respectively), fewer teeth (2.07, 2.08, and 2.48, respectively), higher proportion of severe depressive symptoms (23.4%, 26.2%, and 30.4%, respectively).

As shown in Table S3, higher SOC groups also showed better health status and well-being even if they experienced lighter property damage or housing loss except for drinking alcohol and posttraumatic stress symptoms (PTSS). Among respondents who experienced less severe housing damage, the higher SOC group exhibited a higher proportion of current drinking alcohol compared to the lower SOC group (35.7% vs. 31.6%). And, among respondents who lost their housing, higher SOC group showed a larger prevalence of severe PTSS in comparison with lower SOC group (31.7% vs. 27.9%).

Table 2 shows the results of linear and logistic regression models for the association between the two-way interaction of housing damage with SOC and each outcome, and p-values after Bonferroni correction for multiple testing (0.05/28 outcomes = *p* < 0.002 as the 0.05 threshold). We also calculated E-values and their confidence intervals (Table S4), which is an indication of the minimum strength of association with both the exposure and the outcome that an unmeasured confounder would need to have, conditional on the adjusted covariates, to explain away the observed exposure-outcome relationship. Among respondents who experienced less severe housing damage, higher SOC was inversely associated with some outcomes of worse health and well-being: mental distress (coefficient − 0.29, 95% CI (confidence interval) − 0.39, − 0.19, *p* < 0.01) (each 95% CI is shown in Table S4), depressive symptoms (odds 0.70, 95% CI 0.53, 0.93, *p* = 0.02), poor sleep quality (coefficient − 0.15, 95% CI − 0.25, − 0.04, *p* = 0.01), unhappiness (coefficient − 0.33, 95% CI − 0.43, − 0.23, *p* < 0.01), low expectation of mutual help in the local community (coefficient − 0.17, 95% CI − 0.27, − 0.07, *p* < 0.01), weak community attachment (coefficient − 0.20, 95% CI − 0.30, − 0.11, *p* < 0.01), low frequency of meeting friends (coefficient − 0.11, 95% CI − 0.20, − 0.02, *p* = 0.02), and low frequency of participation in sports clubs (coefficient − 0.10, 95% CI − 0.18, − 0.01, *p* = 0.02). Among these associations, p-values for mental distress, unhappiness, low expectation of mutual help in the local community, and weak community attachment were below the 0.05 threshold after Bonferroni correction for multiple testing (Table S4), and E-values for these outcomes also supported the robustness of associations (ranged from 1.61 to 2.04, Table S4). For example, these E-values suggest that an association of an unmeasured confounding variable and both the exposure and the outcome would need to be at least 1.61 in the risk ratio scale, above and beyond the adjusted covariates, to account for the observed association between SOC and the outcomes.

**Table 2:** Two-way interaction effects of housing damage and sense of coherence on each outcome.

Outcomes	Two-way interactions of housing damage and SOC (ref.: no housing damage*lower SOC)				
	No housing damage * higher SOC	Milder housing damage * lower SOC	Milder housing damage * higher SOC	Housing destruction * lower SOC	Housing destruction * higher SOC
	Coefficient/odds				
Cognitive health					
Cognitive decline diagnosed by physicians, coefficient	0.02	0.09	0.03	0.59***	0.26*
Cognitive decline diagnosed by investigators, coefficient	− 0.01	0.04	− 0.01	0.34***	0.18*
Physical health					
Physical disabilities, coefficient	− 0.03	0.10	0.01	0.49***	0.20
Impaired higher-level IADL, coefficient	0.02	0.08*	0.03	0.44***	0.18
Less remained teeth, coefficient	0.01	0.01	0.01	0.20**	0.21*
Incident of fall, odds	1.01	1.55	0.95	1.60	1.16
Obesity, odds	0.94	0.96	1.18	2.33*	1.59
The number of existing diseases, coefficient	− 0.01	0.11*	0.04	0.01	0.03
Mental health					
Mental distress, coefficient	− 0.43***	0.16***	− 0.29***	0.42***	0.07
Depressive symptoms, odds	0.55***	1.31*	0.70*	2.37**	2.41**
PTSS odds	0.52**	2.10***	1.15	2.95***	3.78***
Poor sleep quality, coefficient	− 0.22***	0.12*	− 0.15**	0.02	− 0.22
Health behavior					
Less daily walking time, coefficient	0.04	0.07	0.05	− 0.01	0.31**
Decreased frequency of going out, odds	0.92	1.27	1.03	1.15	1.02
Current smoking, odds	2.86**	2.35*	2.87**	4.91*	5.93*
Current drinking, odds	1.19	0.83	0.91	1.18	1.14
Subjective well-being					
Low self-rated health, coefficient	− 0.10*	0.11*	− 0.05	− 0.01	0.09
Unhappiness, coefficient	− 0.36***	− 0.01	− 0.33***	0.28*	0.01
Cognitive social capital					
Low expectation of mutual help, coefficient	− 0.11*	− 0.01	− 0.17***	0.10	− 0.02
Less trust to local residents, coefficient	− 0.01	0.01	− 0.07	0.16	0.10
Weak community attachment, coefficient	− 0.10*	− 0.03	− 0.20***	0.63***	0.59***
Social well-being					
Low frequency of meeting friends, coefficient	− 0.04	− 0.04	− 0.11*	− 0.18	− 0.24*
Low frequency of participation in sports clubs, coefficient	− 0.01	− 0.06	− 0.10*	0.08	− 0.03
Low frequency of participation in hobby clubs, coefficient	− 0.04	− 0.04	− 0.05	0.10	− 0.19
Small number of friends meeting, coefficient	0.08	0.06	0.16***	0.19	0.15
Unwillingness to cooperation, coefficient	− 0.14*	0.03	− 0.07	0.11	0.09
Few emotional social support, coefficient	− 0.01	− 0.04	− 0.04	0.33**	0.16
Few instrumental social support, coefficient	0.06	0.05	− 0.02	0.23*	0.08

Results of multivariate liner or logistic regression models adjusting for loss of loved ones during the disaster, sex, age, educational attainment, working status, equivalized household income, divorced/bereavement, living alone, baseline depressive symptoms (≧ 5p of GDS-15), and baseline outcomes.

Coef, Coefficient; SOC, Sense of Coherence.

*p < .05, **p < .01, ***p < .002 (threshold after Bonferroni correction for multiple testing: .05/28 outcomes = .002).

For respondents who experienced more severe damage (complete housing loss), higher SOC was inversely associated with only a low frequency of meeting friends (coefficient − 0.24, 95% CI − 0.47, − 0.02, *p* = 0.04) while the p-value was not below the 0.05 threshold after Bonferroni correction for multiple testing (i.e., *p* < 0.002). For several outcomes, the higher SOC group showed positive associations with worse health and well-being, but the point estimates were smaller than the lower SOC group: cognitive decline assessed by physicians (higher SOC: coefficient 0.26, 95% CI 0.02, 0.50, *p* = 0.03; lower SOC: coefficient 0.59, 95% CI 0.32, 0.86, *p* < 0.01) and trained investigators (higher SOC: coefficient 0.18, 95% CI 0.03, 0.34, *p* = 0.02; lower SOC: coefficient 0.34, 95% CI 0.17, 0.50, *p* < 0.01), and weak community attachment (higher SOC: coefficient 0.59, 95% CI 0.35, 0.83, *p* < 0.01; lower SOC: coefficient 0.63, 95% CI 0.41, 0.84, *p* < 0.01). The p-values for weak community attachment in both lower and higher SOC groups were below the 0.05 threshold after Bonferroni correction (E-value: 2.95 for lower SOC group, 2.81 for higher SOC group; Table S4). In contrast to our expectations, the higher SOC group exhibited larger point estimates in depressive symptoms (lower SOC: Odds 2.37, 95% CI 1.32, 4.24, *p* < 0.01; higher SOC: odds 2.41, 95% CI 1.30, 4.46, *p* = 0.01) and PTSS (lower SOC: Odds 2.95, 95% CI 1.56, 5.56, *p* < 0.01; higher SOC: odds 3.78, 95% CI 2.00, 7.17, *p* < 0.01). The p-values for PTSS in both lower and higher SOC groups were below the 0.05 threshold after Bonferroni correction, and the E-values also supported the robustness of the association (E-value: 5.35 for lower SOC group, 7.02 for higher SOC group; Table S4).

Using a continuous variable of SOC, we examined the interaction effects of SOC and housing damage on the same outcomes in a sensitivity analysis. The results showed a similar pattern to the main analysis (Table S5).

In addition, we implemented another sensitivity analysis using a variable of five levels of housing damage (1 = no damage, 2 = partial damage, 3 = minor, 4 = major, and 5 = complete destruction). The results showed a similar trend to the main analysis. That is, higher SOC tended to protect health and well-being even among respondents with major housing damage (Table S6).

## Discussion

This study demonstrated that higher SOC buffered the impact of less severe housing damage on mental distress, unhappiness, low expectation of mutual help in the local community, and weak community attachment of older survivors in the aftermath of the 2011 earthquake and tsunami, however, higher SOC did not mitigate the impact of housing loss on health and well-being. On the contrary, higher SOC also amplified the impact of housing loss on PTSS.

Most previous studies have measured SOC after traumatic events. Therefore, they cannot rule out the possibility of reverse causation, reporting bias, and survivorship bias. Our study employed a natural experimental design to collect panel data of 3350 older survivors of a natural disaster and suggested that pre-disaster SOC plays a role in mitigating the adverse health consequences of less severe disaster damage. However, we unexpectedly found that higher SOC before the disaster appeared to intensify the impact of hosing destruction on PTSS.

There are plausible reasons for the adverse effect of higher SOC on health and well-being among disaster survivors. The efficacy of stress coping based on SOC rests upon the availability of generalized resistance resources (GRRs) such as social support, financial status, assets (e.g., housing), occupation, and other factors^10^. Victims of severe traumatic events were more likely to lose these resources, thereby facing difficulty in stress coping. In this study, 91% of respondents who experienced housing destruction moved out of their residences after the disaster (Table S7). That is, they lost multiple types of GRRs because of residential relocation. As a result, high SOC prior to the disaster could not play the role of a buffer against the impact of housing loss on health and well-being.

Conservation of resources theory also provides insight into the reason for the adverse effect of higher SOC. This theory postulates that people are motivated to minimize net loss of resources (e.g., housing or money) in stressful life events, and they could be vulnerable if they fail to manage the amount of their resources^17^. Older victims who lost housing may have experienced difficulty in securing a new housing loan due to the absence of collateral assets (e.g. savings). This could ultimately lead to greater mental distress.

Furthermore, a portion of the respondents may have achieved posttraumatic growth (PTG) by the time of the follow-up survey. Despite being under considerable psychological strain, a subset of the survivors can achieve personal growth through struggling with adversities. Posttraumatic growth is a positive psychological change toward traumatic events, referring to five major dimensions: improved relationship with others, increased personal strength, identification of new possibilities, positive spiritual changes, and increased appreciation of life^18^. A meta-analysis demonstrated that adult survivors exposed to earthquakes tend to have a moderate level of PTG^19^. Research has suggested that SOC is positively associated with PTG, because people who have higher SOC are capable of coping with stressful life events^20,21^. We did not measure PTG in the follow-up survey, however, previous studies also suggested that PTSS is positively associated with PTG because a certain level of struggle is necessary for achieving PTG^22^. Therefore, the amplified PTSS among respondents with higher SOC might have implied that their PTG was raised by the time of the follow-up survey.

Traumatic stress studies have separately considered the single-incident traumatic event (e.g., traffic accident) known as type I trauma and persistent trauma exposures (e.g., interpersonal conflicts), also known as type II trauma^23^. And, they also have suggested that type II trauma exposures might have stronger associations with PTSS severity than type I traumatic experiences^24^. Our respondents have reported prolonged suffering owing to the disaster. For example, some respondents who could not recoup their own housing exhibited a higher risk of cognitive decline six years after the disaster^25^. Therefore, traumatic experiences in massive natural disasters could be long-term stressors (i.e., type II trauma) for at least some victims who suffer severe damage.

A major strength of the present study is the availability of information predating the disaster about SOC and confounders. Our design was therefore able to effectively address the problem of recall bias in most studies conducted in post-disaster settings. A second strength is the usage of an outcome-wide approach to evaluate the role of SOC on health and well-being among disaster survivors. This approach enables us to rule out the possibility of false discovery.

A limitation of this study was the possibility of selection bias due to 59% response rate at the baseline survey. Nonetheless, this response rate is quite comparable to similar surveys involving community-dwelling residents^26^. In addition, we confirmed that the demographic profile of our participants at baseline was similar to the rest of the Iwanuma residents aged 65 years or older (Table S1). Furthermore, the participation rates of our follow-up surveys were quite high (82.1%). Another limitation is unvalidated measurements of SOC. However, we confirmed the psychometric validity of our six items through a confirmatory factor analysis (Figure S1).

In conclusion, our study demonstrated that higher SOC could exert a protective effect on the mental distress, unhappiness, low expectation of mutual help in the local community, and weak community attachment of older disaster survivors, particularly among those who experienced less severe forms of disaster trauma. On the other hand, our study also reveals that higher SOC can have a deleterious effect on PTSS among those who experienced more severe forms of property damage. Our findings suggested that SOC functions to cope with stressful life events if enough GRR is still available for victims after traumatic events, however, SOC may not be protective for health and well-being under conditions of resource deficiency. To mitigate any detrimental effects of SOC, recovery efforts after disasters need to prioritize prompt rehousing of dislocated individuals together with financial aid and appropriate social support (e.g., social services).

## Methods

### Study participants

The Japan Gerontological Evaluation Study (JAGES) was established in 2010 as a nationwide sample of older adults aged 65 years or older. One of the field sites of the JAGES cohort is based in the city of Iwanuma (total population 44,187 in 2010). We mailed questionnaires to every resident aged 65 years or older in August 2010 (n = 8576), using the official residential register of Iwanuma. The survey inquired about personal characteristics, sense of coherence, and health status. The response rate was 59.0% (n = 5058), which is comparable to other surveys of community-dwelling residents^26^.

The earthquake and tsunami occurred on March 11th 2011, seven months after the baseline survey was completed. Iwanuma City is a coastal municipality located approximately 80 km west of the earthquake epicenter, so it was in the direct line of the tsunami. That disaster killed 180 residents, damaged 5542 housing units, and inundated 48% of the land area (Fig. 1).

**Figure 2:**
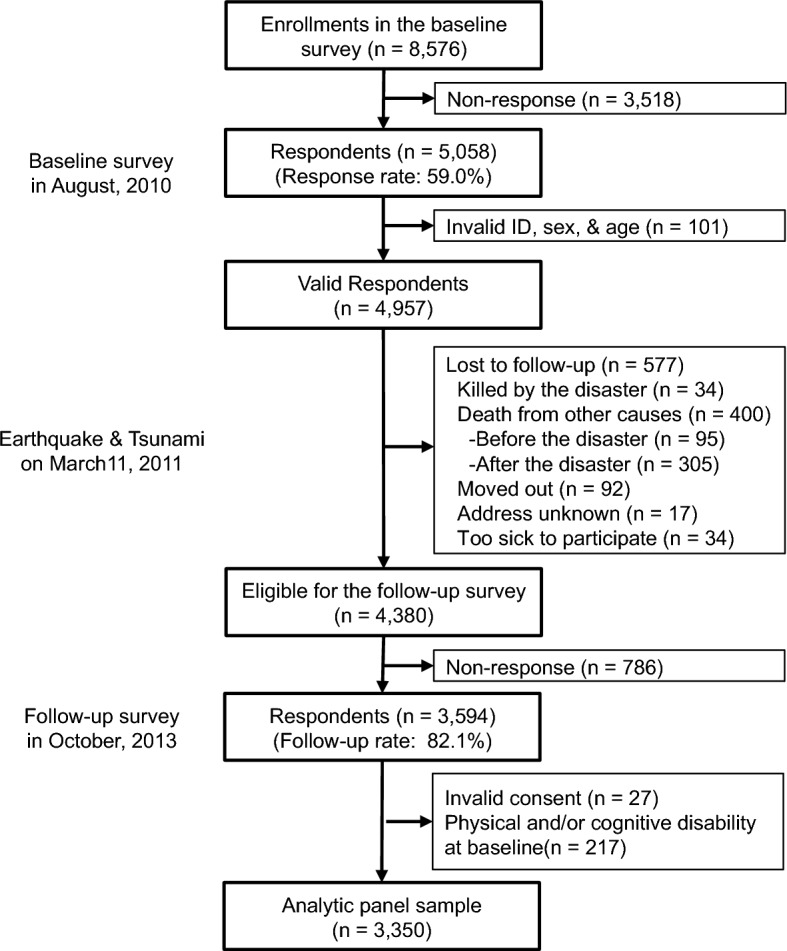
Participant flow in this study.

We conducted a follow-up survey of survivors approximately 2.5 years after the disaster (starting October 2013). The survey gathered information about personal experiences during the disaster and updated information about health status. The detailed flowchart of the analytic sample is presented in Fig. 2. Of the 4380 eligible participants from the baseline survey, we managed to recontact 3594 individuals (participation rate: 82.1%). We finally obtained 3350 participants after excluding 27 individuals who incompletely signed informed consent forms and 217 individuals who had physical and/or cognitive disabilities at the baseline.

The study was approved by the human subjects committee of the Harvard T.H. Chan School of Public Health (23143) as well as the human subjects committees of Tohoku University (24-29), Nihon Fukushi University (13-17), and Chiba University (235). Informed consent was obtained at the time of survey collection. All methods were performed in accordance with the relevant guidelines and regulations.

### Outcome variable

Our outcomes of interest were 28 variables of health and well-being in the follow-up survey^15^. We categorized these outcomes into 7 domains: (1) cognitive health (levels of cognitive decline assessed by physicians and trained investigators); (2) physical health (levels of physical disability assessed by trained investigators, and impaired higher-level of instrumental activities of daily living, less remained teeth, incident of fall, obesity (BMI ≧ 25), and the number of existing diseases); (3) mental health (mental distress, depressive symptoms, PTSS, and poor sleep quality); (4) health behavior (less daily walking time, decreased frequency of going out in the past year, current smoking, and current drinking alcohol); (5) subjective well-being (low self-related health and unhappiness); (6) cognitive social capital (low expectation of mutual help in the local community, less trust to local residents, and weak community attachment); and (7) social well-being (low frequency of meeting friends, low frequency of participation in sports clubs, low frequency of participation in hobby clubs, small number of friends meeting in the past month, unwillingness to cooperate with the local community, few emotional social support, and few instrumental social support).

*(1) Cognitive health* The level of cognitive disability was assessed by a standardized in-home assessment under the Japanese Long-Term Care Insurance (LTCI) scheme established in 2000^27^. Registration in this LTCI scheme is mandatory, and each applicant requesting long-term care is assessed for eligibility to receive services (e.g., home help) by a trained investigator dispatched from the certification committee in each municipality. During the home visit, each individual is assessed with regard to their cognitive function (e.g., short-term memory, orientation, and communication) and mental and behavioral disorders (e.g., delusions of persecution and confabulation) using a standardized protocol. Following the assessment, the applicants requesting long-term care are classified into one of seven levels (1: Suffering some cognitive deficits, but otherwise almost completely independent to 7: Needs constant treatment in a specialized medical facility) according to the severity of their cognitive disability. The index of cognitive decline is strongly correlated with the Mini-Mental State Examination (Spearman’s rank correlation ρ = − 0.73, *p* < 0.01)^28^, and level I of the cognitive disability scale has been demonstrated to correspond with a 0.5-point rating on the Clinical Dementia Rating scale (both specificity and sensitivity were 0.88)^29^.

The committee also asks a panel of physicians to independently assess the cognitive disability level of applicants to determine the care requirements of the applicants^30^. We used the result of physician’s assessment for cognitive decline as well.

*(2) Physical health* In the LTCI scheme, the trained investigators assess applicants’ activities of daily living and instrumental activities of daily living, and classify the applicants into one of eight levels (1: Suffers from some form of disability but is mostly autonomous in daily life and can manage outings alone using public transportations to 8: Spends the whole day in bed and requires assistance in waste elimination, meals, changing clothes, and even turning over).

Higher-level IADL was measured by TMIG-IC (Tokyo Metropolitan Institute of Gerontology Index of Competence), which consists of 13 items asking about physical and cognitive performances^31^. We reversed the IADL score whose higher scores indicate lower ability to perform these instrumental activities.

Participants were asked about the number of remained teeth in the surveys using the following choices: 1 = 20 and more, 2 = 10 to 19, 3 = 1 to 9, and 4 = 0. The higher score means fewer remained teeth.

We also asked them about experiences of falls in the past year. They chose either one from three choices: 1 = many times, 2 = once, and 3 = none. And, we created a binary variable (1 = many times and 0 = once or none).

We calculated BMI using self-reported height and weight in both waves (2010 and 2013). The accuracy of self-reported BMI has been previously demonstrated in a Japanese older population, by comparing with physical measurements of BMI^32^. We categorized BMI into two categories, according to World Health Organization classification for Asian populations^33^: < 25.0 (non-obese) and ≥ 25.0 (obese).

Participants also answered whether they have any kinds of diseases among following choices: 1 = cancer, 2 = heart disease (including arrhythmia), 3 = stroke, 4 = high blood pressure, 5 = diabetes (including a mild form), 6 = obesity, 7 = hyperlipidemia, 8 = osteoporosis, 9 = joint disease/neuralgia, 10 = injury/fracture, 11 = respiratory disease, 12 = gastrointestinal disease, 13 = liver disease, 14 = mental disease, 15 = difficulty swallowing, 16 = impaired vision, 17 = Impaired hearing, 18 = elimination problems (including incontinence, frequent urination, difficulty in starting urination, leaking of urine, etc.), and 19 = sleep problem. We summed up the number of diseases that respondents chose.

*(3) Mental health* We used the Japanese version of Kessler Psychological Distress-6 (K6) to assess the level of mental distress^34^.

Depressive symptoms were measured by the Japanese version of Geriatric Depression Scale-15 (GDS-15)^35^. The score was categorized into lower (four points and under) versus higher (five points and over) risks^36^.

PTSS was assessed using the Screening Questionnaire for Disaster-Related Mental Health^37^, originally developed and psychometrically validated by a team of Japanese researchers in the aftermath of the Hanshin-Awaji earthquake in 1995. The instrument was specifically designed for use in older individuals and has been psychometrically validated against the Japanese-language version of the Clinician-Administered PTSD Scale^38^, as well as the Impact of Event Scale-Revised, Japanese version^39^. The scale is made up of 9 items, with the following predefined cutoff points for PTSS: slightly affected (0–3 points), moderately affected (4–5 points), and severely affected (6–9 points). In the present study, we categorized the response scores into two risk levels (1 = severely affected, 0 = moderately affected or slightly affected).

Poor sleep quality was measured by using the question “How do you evaluate your sleep quality over the past month?”. Respondents chose an answer from four choices: 1 = very good, 2 = good, 3 = poor, and 4 = very poor.

*(4) Health behavior* Less daily walking time was measured using the question “How long do you walk a day on average?”. Respondents chose an answer from four choices (1 = less than 30 min, 2 = 30 to 59 min, 3 = 60 to 89 min, and 4 = 90 min or more). We reversed the score to create the scale in which higher scores indicate a shorter time of walking.

We also asked respondents whether their frequency of going out has decreased since last year. They chose 1 = yes or 0 = no.

Respondents also answered their status of smoking and drinking alcohol. They chose an appropriate answer for smoking from four choices: 1 = I have never smoked, 2 = I stopped smoking 5 or more years ago, 3 = I stopped smoking within the past 4 years, and 4 = I am currently a smoker. We created a binary variable (1 = I am currently a smoker and 0 = I have never smoked, I stopped smoking 5 or more years ago, or I stopped smoking within the past 4 years). They also answered their habit of drinking alcohol (1 = yes, 2 = I used to drink, or 3 = no). Their answer was categorized into two groups: 1 = yes and 0 = I used to drink or no.

*(5) Subjective well-being* We asked participants about their self-rated health using the question “How is your current health status?”. They rated their self-rated health on a 4-point Likert scale: 1 = excellent to 4 = poor, which higher scores mean worse health status.

They also rated their subjective happiness on a 10-point Likert scale. We reversed the score to create a scale of unhappiness.

*(6) Cognitive social capital* Respondents were asked about perceptions of expectation for mutual help among local residents, trust in the community, and community attachment, and chose their answer on a 5-point Likert scale (1 = very to 5 = not at all), with higher scores representing lower levels of cognitive social capital.

*(7) Social well-being* We asked participants about the frequency of meeting friends, and they chose an appropriate answer on a 6-point Likert scale: 1 = almost every day to 6 = rarely, with higher scores indicating lower frequency.

We also asked them the frequency of participating in sports and hobby clubs, using a 6-point Likert scale: 1 = almost every day to 6 = never.

The number of friends or acquaintances was also measured using the question “How many friends/acquaintances have you seen over the past month? Count the same person as one, no matter how many times you have seen him/her.”. They answered on a 5-point Likert scale: 1 = none to 6 = 10 or more. We reversed the score representing a small number of friends.

Participants also asked about willingness to cooperate with the local community using the question “Do you agree with making it a rule to offer half a day for the interests of the whole area but not for your own interests?”. They chose an appropriate answer from three choices: 1 = agree, 2 = Neutral, or 3 = I disagree. That is, higher scores indicate their unwillingness.

In the survey, we measured respondents’ receiving emotional/instrumental social support. They were asked whether they have someone who listens to your concerns or complaints (emotional support) and someone who looks after you when you are sick and confined to a bed for a few days (instrumental support). They answered who provide social support from the following choices: 1 = spouse, 2 = children living together, 3 = Children or relatives living apart, 4 = neighbor, 5 = friend, and 6 = other. We summed up the number of providers and reversed scores to create variables that represent little emotional/instrumental support.

The details of these outcome variables were also provided in Table S8.

### Explanatory variables

One of the primary explanatory variables is SOC at the baseline. We asked participants about three dimensions of SOC: comprehensibility, manageability, and meaningfulness^10^. Comprehensibility is a sense that every life event happening to oneself is understandable and could be foreseen because the world is ordered and structured. This dimension was measured using the following questions: “Does your feeling or thinking get very confused? (1: very often, 5: never)”; and “Do you experience undesired emotions? (1: very often, 5: never)”. Manageability is a belief that there is own competence to cope effectively with problems in one’s life. Participants were asked: “Do you feel you are treated unfairly? (1: very often, 5: never)”; and “Do you lose confidence in your ability to keep self-control?” (1: very often, 5: never)”. Meaningfulness is a belief that solving difficult problems is worth engaging and reasonable to spend own time and effort. We asked participants this dimension using the following questions: “Do you feel what you do every day has little meaning to you? (1: very often, 5: never)”; and “What you do every day… (1: Gives you pleasure and satisfaction, 5: Gives you no pleasure or satisfaction–reverse code measurement). We calculated the arithmetical mean of the six items to create the total SOC score (mean 3.69, standard deviation (SD) 0.70) because of good internal consistency (Cronbach’s α = 0.83). And then, we created a binary variable that categorized participants into two groups using a median split (median 3.67).

Another primary exposure variable of interest is housing damage in the disaster. Our previous outcome-wide study demonstrated that housing damage is a unique predictor for various types of outcomes and well-being^15^. Respondents retrospectively answered housing damage assessed by property inspectors, which was classified into five levels (1 = no damage, 2 = partial damage, 3 = minor, 4 = major, and 5 = complete destruction). We categorized the level of housing damage into three groups (1 = no damage, 2 = milder housing damage, and 3 = complete destruction).

Respondents also reported loss of loved ones (close friends and/or relatives) during the disaster.

### Covariates

According to previous studies^25,40, 41^, we selected several demographic variables as potential confounding variables for the association of disaster experiences and SOC with cognitive disability: age, sex, income, educational attainment, divorce or bereavement, working status, living alone, and depressive symptoms at the baseline.

We also selected 18 pre-exposure outcomes as confounders. The baseline cognitive decline and physical disability were not considered in the analysis because respondents who had physical and/or cognitive disabilities at the baseline were excluded from our analytic sample. Furthermore, we could not control for mental distress, PTSS, poor sleep quality, and subjective unhappiness in the analysis because of the lack of measurements in the baseline questionnaire.

Depressive symptoms at the baseline and follow-up survey were measured by the Geriatric Depression Scale-15, and categorized into lower risk (4 points and under) versus higher risk (5 points and over)^36^. Household income was equivalized by dividing the gross income by the square root of the number of household members^42^.

### Statistical analysis

We utilized an outcome-wide approach that enables a holistic assessment of the relationship between a single exposure and a wide range of outcomes. We employed this approach to examine whether baseline SOC modified the association between housing damage and the 28 outcomes in the follow-up survey. Logistic and linear regression models were implemented to estimate coefficients or odds ratios for each outcome. All continuous outcomes were standardized (mean = 0, standard deviation (SD) = 1), so that the effect estimates can be interpreted as a SD change in the outcome variable. We used a Bonferroni correction to account for multiple testing and used 0.05/28 outcomes = *p* < 0.002 as a threshold for statistical significance. To evaluate the robustness of our effect estimates to unmeasured confounding, we calculated E-values and their limit of confidence interval for each exposure–outcome association^15^. E-value indicates the effect size that an unmeasured confounder would need to have on both the exposure and the outcome to explain away the observed exposure-outcome relationship, above and beyond the observed covariates.

We also implemented multiple imputations using the Markov chain Monte Carlo method that assumes missingness at random for the explanatory variables and covariates. Fifty datasets were created and combined each result of analyses using the Stata command “mi estimate.” All analyses were performed using STATA version 17.0 (STATA Corp LP, College Station, TX, USA).

### Supplementary Information

Supplementary Information.

## Data Availability

All data needed to evaluate the conclusions in the paper are present in the paper and/or the Supplementary Materials. The JAGES data used in this study will be made available upon request, as per NIH data access policies. The authors require the applicant to submit an analysis proposal to be reviewed by an internal JAGES committee to avoid duplication. Confidentiality concerns prevent us from depositing our data in a public repository. Authors requesting access to the Iwanuma data need to contact the principal investigator of the parent cohort (K.K.) and the Iwanuma sub-study principal investigator (I.K.) in writing. Proposals submitted by outside investigators will be discussed during the monthly investigators’ meeting to ensure that there is no overlap with ongoing analyses. If approval to access the data is granted, the JAGES researchers will request the outside investigator to help financially support our data manager’s time to prepare the data for outside use.

## Abbreviations

JPY: Japanese Yen
SOC: Sense of Coherence
GDS-15: Geriatric Depression Scale-15
