# Supplementary material for: Association between sense of coherence and health and well-being among older survivors of a natural disaster: a prospective outcome-wide study
Source: Sci Rep. 2023 Sep 29;13:16385. doi: 10.1038/s41598-023-43672-z (PMC10542327; doi:10.1038/s41598-023-43672-z)
Supplement: Supplementary file 1 — Supplementary Information. [file 41598_2023_43672_MOESM1_ESM.docx]

**Supplementary Information**

Association between sense of coherence and health and well-being among older survivors of a natural disaster: a prospective outcome-wide study

**Authors:** Hiroyuki Hikichi, PhD; Koichiro Shiba, PhD; Jun Aida, DDS, PhD; Katsunori Kondo, MD, PhD; and Ichiro Kawachi, MD, PhD

**Corresponding author:** Hiroyuki Hikichi, PhD

**Email:** hikichi@med.kitasato-u.ac.jp

Table S1. Baseline characteristics of the analytic sample and whole older population in Iwanuma City

Figure S1. A result of confirmatory factor analysis for sense of coherence

Table S2. Descriptive statistics of pre-exposure outcomes by leves of housing damage

Table S3. Descriptive statistics of outcomes by levels of SOC and housing damage

Table S4. Two-way interaction effects of housing damage and sense of coherence on each outcome

Table S5. Two-way interaction effects of housing damage and a continuous variable of sense of coherence on each outcome

Table S6. Two-way interaction effects of housing damage (five levels) and a binary variable of sense of coherence on each outcome

Table S7. Association between levels of housing damage and residential relocation

Table S8. Definitions of outcomes

Table S1. Baseline characteristics of the analytic sample and whole older population in Iwanuma City

|  | Analytic sample at baseline | | Whole older population of Iwanuma City from local census in 2010 | |
| --- | --- | --- | --- | --- |
|  | n | % | n | % |
| Sex |  |  |  |  |
| Male | 1,493 | 44.6 | 3,735 | 42.8 |
| Female | 1,857 | 55.4 | 4,988 | 57.2 |
| Age |  |  |  |  |
| 65-74 year | 2,080 | 62.1 | 4,523 | 51.8 |
| 75-84 year | 1,109 | 33.1 | 3,050 | 35.0 |
| 85 year and over | 161 | 4.8 | 1,150 | 13.2 |
| Marital status ^a^ |  |  |  |  |
| Married | 2,421 | 74.8 | 5,618 | 64.7 |
| Unmarried, widowed, and divorced | 816 | 25.2 | 3,068 | 35.3 |
| Working status ^b^ |  |  |  |  |
| Working | 550 | 18.6 | 1,493 | 17.2 |
| Not working | 2,412 | 81.4 | 7,169 | 82.8 |

a, b Sample size is not the same with the overall sample (n = 3,350) due to missingness

Figure S1. A result of a confirmatory factor analysis for sense of coherence

Note: The goodness of fit indices: comparative fit index (CFI) 0.998, root-mean-square error of approximation (RMSEA) 0.023, and standardized-root-mean-square residual (SRMA) 0.011

Table S2. Descriptive statistics of pre-exposure outcomes by leves of housing damage

|  | No damage  (n = 1,334) | Milder housing damage  (n = 1,778) | Housing loss  (n = 148) |
| --- | --- | --- | --- |
|  | n (%)/mean (SD) | | |
| Outcomes at the baseline |  |  |  |
| Cogitive health |  |  |  |
| Levels of cognitive decline assessed by medical doctors, mean (SD) ^a^ | - | - | - |
| Levels of cognitive decline assessed by trained investigators, mean (SD) ^a^ | - | - | - |
| Physical health |  |  |  |
| Levels of physical disability, mean (SD) ^a^ | - | - | - |
| Impaired higher-level IADL (TMIG-IC), mean (SD) | 1.07 (1.66) | 1.11 (1.83) | 1.58 (2.50) |
| Missing, n(%) | 90 (6.7) | 99 (12.7) | 11 (7.4) |
| Less remained teeth, mean (SD) | 2.07 (1.02) | 2.08 (1.00) | 2.48 (1.12) |
| Missing, n(%) | 20 (1.5) | 30 (1.7) | 3 (2.0) |
| Incident of fall, n (%) | 72 (5.4) | 105 (5.9) | 11 (7.4) |
| Missing, n(%) | 22 (1.7) | 23 (1.3) | 4 (2.7) |
| Obesity, n (%) | 345 (25.9) | 494 (27.8) | 38 (25.7) |
| Missing, n(%) | 69 (5.2) | 103 (5.8) | 19 (12.8) |
| The number of existing diseases, mean (SD) | 1.94 (1.31) | 1.96 (1.36) | 1.92 (1.28) |
| Missing, n(%) | 334 (25.0) | 422 (23.7) | 37 (25.0) |
| Mental health |  |  |  |
| Mental distress (K6), mean (SD) ^b^ | - | - | - |
| Missing, n(%) | - | - | - |
| Depressive symptoms (>=5 points of GDS-15 score), n (%) | 312 (23.4) | 466 (26.2) | 45 (30.4) |
| Missing, n(%) | 167 (12.5) | 236 (13.3) | 26 (17.6) |
| Posttraumatic stress symptoms (>=6 points of SQD score), n (%) ^b^ | - | - | - |
| Missing, n(%) | - | - | - |
| Poor sleep quality, mean (SD) ^b^ | - | - | - |
| Missing, n(%) | - | - | - |
| Health behavior |  |  |  |
| Less daily walking time, mean (SD) | 2.95 (1.00) | 2.93 (1.03) | 2.98 (1.10) |
| Missing, n(%) | 48 (3.6) | 74 (4.2) | 9 (6.1) |
| Decreased frequency of going out in the past year, n (%) | 326 (24.4) | 493 (27.7) | 34 (23.0) |
| Missing, n(%) | 25 (1.9) | 37 (2.1) | 4 (2.7) |
| Current smoking, n (%) | 139 (10.4) | 185 (10.4) | 19 (12.8) |
| Missing, n(%) | 95 (7.1) | 145 (8.2) | 20 (13.5) |
| Current drinking, n (%) | 506 (37.9) | 668 (37.6) | 45 (30.4) |
| Missing, n(%) | 23 (1.7) | 35 (2) | 9 (6.1) |
| Subjective well-being |  |  |  |
| Low self-reated health, mean (SD) | 2.02 (0.59) | 2.08 (0.58) | 2.08 (0.74) |
| Missing, n(%) | 20 (1.5) | 34 (1.9) | 6 (4.1) |
| Subjective unhappiness, mean (SD) ^b^ | - | - | - |
| Missing, n(%) | - | - | - |
| Social connectedness |  |  |  |
| Low expectation for mutual help in the local community, mean (SD) | 2.48 (0.8) | 2.43 (0.82) | 2.49 (0.86) |
| Missing, n(%) | 40 (3.0) | 40 (2.2) | 10 (6.8) |
| Less trust to local residents, mean (SD) | 2.25 (0.74) | 2.22 (0.76) | 2.29 (0.89) |
| Missing, n(%) | 17 (2.0) | 26 (1.5) | 9 (6.1) |
| Weak community attachment, mean (SD) | 2.04 (0.82) | 1.96 (0.81) | 1.93 (0.92) |
| Missing, n(%) | 32 (2.4) | 32 (1.8) | 6 (4.1) |
| Low frequency of meeting friends, mean (SD) | 3.34 (1.46) | 3.15 (1.44) | 3.05 (1.63) |
| Missing, n(%) | 52 (3.9) | 52 (2.9) | 12 (8.1) |
| Low frequency of participation in sports clubs, mean (SD) | 5.13 (1.46) | 5.03 (1.47) | 5.56 (1.08) |
| Missing, n(%) | 194 (14.5) | 266 (15.0) | 45 (30.4) |
| Low frequency of participation in hobby clubs, mean (SD) | 4.78 (1.44) | 4.60 (1.50) | 5.16 (1.21) |
| Missing, n(%) | 167 (12.5) | 242 (13.6) | 42 (28.4) |
| Small number of friends meeting in the past month, mean (SD) | 3.55 (1.27) | 3.69 (1.25) | 3.59 (1.30) |
| Missing, n(%) | 62 (4.6) | 62 (3.5) | 13 (8.8) |
| Unwillingness to cooperate with the local community, mean (SD) | 1.52 (0.56) | 1.53 (0.57) | 1.44 (0.57) |
| Missing, n(%) | 74 (5.5) | 87 (4.9) | 14 (9.5) |
| Few emotional social support, mean (SD) | 3.44 (0.97) | 3.33 (1.03) | 3.37 (1.12) |
| Missing, n(%) | 42 (3.1) | 69 (3.9) | 9 (6.1) |
| Few instrumental social support, mean (SD) | 3.63 (0.73) | 3.56 (0.75) | 3.60 (0.77) |
| Missing, n(%) | 28 (2.1) | 42 (2.4) | 7 (4.7) |

Abbreviation: JPY, Japanese Yen; GDS-15, Geriatric Depression Scale-15; TMIG-IC, Tokyo Metropolitan Institute of Gerontology Index of Competence; SQD, Screening Questionnaire for Disaster mental health

^a^ Respondents who had physical and/or cognitive disabilities were excluded from the analytic samples.

^b^ These variables were not measured in the baseline survey.

Table S3. Descriptive statistics of outcomes by levels of SOC and housing damage

|  | No housing damage and low SOC  (n = 565) | No housing damage and high SOC  (n = 654) | Lighter housing damage and low SOC  (n = 827) | Lighter housing damage and high SOC  (n = 801) | Housing destruction and low SOC (n = 76) | Housing destruction and high SOC (n = 46) |
| --- | --- | --- | --- | --- | --- | --- |
| Outcomes in the follow-up survey | mean/n (SD/%) | | | | | |
| Cogitive health |  |  |  |  |  |  |
| Levels of cognitive decline assessed by medical doctors, mean (SD) | 1.10 (0.58) | 1.07 (0.48) | 1.15 (0.65) | 1.07 (0.48) | 1.42 (1.17) | 1.35 (1.22) |
| Levels of cognitive decline assessed by trained investigators, mean (SD) | 1.15 (0.62) | 1.09 (0.49) | 1.18 (0.71) | 1.09 (0.48) | 1.45 (1.11) | 1.37 (1.14) |
| Physical health |  |  |  |  |  |  |
| Levels of physical disability, mean (SD) | 1.27 (0.95) | 1.17 (0.79) | 1.37 (1.16) | 1.19 (0.87) | 1.7 (1.57) | 1.48 (1.46) |
| Impaired higher-level IADL (TMIG-IC), mean (SD) | 1.78 (2.33) | 1.20 (2.05) | 1.97 (2.85) | 1.19 (2.02) | 3.29 (3.59) | 2.21 (3.44) |
| Less remained teeth, mean (SD) | 2.16 (1.35) | 1.98 (1.33) | 2.16 (1.38) | 1.94 (1.21) | 2.84 (1.60) | 2.56 (1.48) |
| Incident of fall, n (%) | 32 (5.7) | 26 (4.0) | 71 (8.6) | 29 (3.6) | 7 (9.3) | 0 (0) |
| Obesity, n (%) | 160 (29.1) | 150 (23.5) | 220 (27.2) | 238 (30.3) | 32 (43.2) | 13 (28.9) |
| The number of existing diseases, mean (SD) | 1.73 (1.33) | 1.54 (1.29) | 1.87 (1.42) | 1.61 (1.25) | 1.68 (1.21) | 1.65 (1.25) |
| Mental health |  |  |  |  |  |  |
| Mental distress (K6), mean (SD) | 5.06 (4.21) | 2.23 (2.93) | 5.87 (4.46) | 2.98 (3.53) | 7.17 (4.49) | 4.63 (4.52) |
| Depressive symptoms (>=5 points of GDS-15 score), n (%) | 205 (41.7) | 71 (12.2) | 344 (46.9) | 124 (16.7) | 35 (54.7) | 15 (39.5) |
| Posttraumatic stress symptoms (>=6 points of SQD score), n (%) | 50 (9.4) | 22 (3.5) | 154 (19.3) | 55 (7.1) | 19 (27.9) | 13 (31.7) |
| Poor sleep quality, mean (SD) | 2.29 (0.68) | 2.06 (0.59) | 2.38 (0.67) | 2.1 (0.61) | 2.28 (0.72) | 2.04 (0.56) |
| Health behavior |  |  |  |  |  |  |
| Less daily walking time, mean (SD) | 2.91 (1.00) | 2.8 (1.02) | 2.96 (1.03) | 2.8 (1.05) | 2.86 (1.14) | 3.2 (0.85) |
| Decreased frequency of going out in the past year, n (%) | 148 (26.7) | 116 (18.2) | 262 (32.4) | 148 (18.9) | 19 (26.0) | 11 (24.4) |
| Current smoking, n (%) | 31 (5.5) | 58 (8.9) | 73 (8.8) | 64 (8.0) | 12 (16.0) | 6 (13.3) |
| Current drinking, n (%) | 188 (33.5) | 236 (36.2) | 260 (31.6) | 284 (35.7) | 25 (32.9) | 12 (26.7) |
| Subjective well-being |  |  |  |  |  |  |
| Low self-reated health, mean (SD) | 2.2 (0.65) | 1.98 (0.57) | 2.29 (0.65) | 2.03 (0.6) | 2.14 (0.65) | 2.02 (0.61) |
| Subjective unhappiness, mean (SD) | 4.4 (1.81) | 3.16 (1.53) | 4.40 (1.81) | 3.31 (1.63) | 4.9 (2.08) | 3.88 (1.90) |
| Social connectedness |  |  |  |  |  |  |
| Low expectation for mutual help in the local community, mean (SD) | 2.57 (0.82) | 2.37 (0.74) | 2.56 (0.82) | 2.30 (0.71) | 2.65 (0.86) | 2.61 (0.87) |
| Less trust to local residents, mean (SD) | 2.34 (0.76) | 2.16 (0.64) | 2.32 (0.72) | 2.13 (0.62) | 2.45 (0.77) | 2.27 (0.59) |
| Weak community attachment, mean (SD) | 2.22 (0.84) | 1.90 (0.73) | 2.15 (0.82) | 1.81 (0.65) | 2.68 (1.11) | 2.49 (1.10) |
| Low frequency of meeting friends, mean (SD) | 3.58 (1.53) | 3.3 (1.53) | 3.4 (1.51) | 3.13 (1.52) | 3.17 (1.52) | 2.84 (1.73) |
| Low frequency of participation in sports clubs, mean (SD) | 5.36 (1.29) | 5.09 (1.50) | 5.22 (1.40) | 4.87 (1.59) | 5.68 (1.02) | 5.55 (1.17) |
| Low frequency of participation in hobby clubs, mean (SD) | 5.25 (1.30) | 4.91 (1.46) | 5.07 (1.33) | 4.84 (1.48) | 5.59 (1.01) | 5.02 (1.45) |
| Small number of friends meeting in the past month, mean (SD) | 3.19 (1.33) | 3.59 (1.34) | 3.38 (1.30) | 3.76 (1.31) | 3.44 (1.38) | 3.47 (1.46) |
| Unwillingness to cooperate with the local community, mean (SD) | 1.70 (0.57) | 1.52 (0.56) | 1.72 (0.57) | 1.55 (0.57) | 1.74 (0.58) | 1.56 (0.55) |
| Few emotional social support, mean (SD) | 4.12 (1.23) | 3.9 (1.29) | 3.98 (1.25) | 3.79 (1.32) | 4.53 (1.22) | 3.80 (1.49) |
| Few instrumental social support, mean (SD) | 4.41 (0.93) | 4.30 (0.89) | 4.40 (0.94) | 4.19 (0.99) | 4.59 (1.06) | 4.28 (0.91) |

Abbreviation: JPY, Japanese Yen; GDS-15, Geriatric Depression Scale-15; TMIG-IC, Tokyo Metropolitan Institute of Gerontology Index of Competence; SQD, Screening Questionnaire for Disaster mental health

Table S4. Two-way interaction effects of housing damage and sense of coherence on each outcome

| Outcome | Explanatory variables | Coef | Odds | 95% CI | | p |  | E-value | |
| --- | --- | --- | --- | --- | --- | --- | --- | --- | --- |
|  | Housing damage * baseline SOC (ref.: no damage and lower SOC) |  |  |  |  |  |  | effect estimate | CI limit |
| Cognitive health |  |  |  |  |  |  |  |  |  |
| Levels of cognitive decline | No housing damage and higher SOC | 0.02 |  | -0.09 | 0.13 | .70 |  | 1.16 | 1.00 |
| assessed by physicians | Milder housing damage and lower SOC | 0.09 |  | -0.02 | 0.19 | .11 |  | 1.38 | 1.11 |
|  | Milder housing damage and higher SOC | 0.03 |  | -0.08 | 0.13 | .63 |  | 1.20 | 1.00 |
|  | Housing destruction and lower SOC | 0.59 |  | 0.32 | 0.86 | < .002 | *** | 2.81 | 2.22 |
|  | Housing destruction and higher SOC | 0.26 |  | 0.02 | 0.50 | .03 | * | 1.85 | 1.08 |
| Levels of cognitive decline | No housing damage and higher SOC | -0.01 |  | -0.07 | 0.07 | .99 |  | 1.11 | 1.00 |
| assessed by trained investigators | Milder housing damage and lower SOC | 0.04 |  | -0.03 | 0.11 | .23 |  | 1.23 | 1.00 |
|  | Milder housing damage and higher SOC | -0.01 |  | -0.08 | 0.06 | .77 |  | 1.11 | 1.00 |
|  | Housing destruction and lower SOC | 0.34 |  | 0.17 | 0.50 | < .002 | *** | 2.07 | 1.54 |
|  | Housing destruction and higher SOC | 0.18 |  | 0.03 | 0.34 | .02 | * | 1.64 | 1.00 |
| Physical health |  |  |  |  |  |  |  |  |  |
| Levels of physical disabilities | No housing damage and higher SOC | -0.03 |  | -0.14 | 0.08 | .57 |  | 1.20 | 1.00 |
|  | Milder housing damage and lower SOC | 0.10 |  | 0.00 | 0.21 | .05 |  | 1.42 | 1.16 |
|  | Milder housing damage and higher SOC | 0.01 |  | -0.10 | 0.12 | .86 |  | 1.11 | 1.00 |
|  | Housing destruction and lower SOC | 0.49 |  | 0.24 | 0.75 | < .002 | *** | 2.50 | 1.94 |
|  | Housing destruction and higher SOC | 0.20 |  | -0.05 | 0.46 | .12 |  | 1.69 | 1.00 |
| Impaired higher-level IADL | No housing damage and higher SOC | 0.02 |  | -0.07 | 0.10 | .69 |  | 1.16 | 1.00 |
|  | Milder housing damage and lower SOC | 0.08 |  | 0.00 | 0.17 | .04 | * | 1.26 | 1.04 |
|  | Milder housing damage and higher SOC | 0.03 |  | -0.05 | 0.11 | .45 |  | 1.20 | 1.00 |
|  | Housing destruction and lower SOC | 0.44 |  | 0.26 | 0.63 | < .002 | *** | 2.35 | 1.81 |
|  | Housing destruction and higher SOC | 0.18 |  | -0.01 | 0.37 | .06 |  | 1.64 | 1.00 |
| Less remained teeth | No housing damage and higher SOC | 0.01 |  | -0.05 | 0.08 | .68 |  | 1.11 | 1.00 |
|  | Milder housing damage and lower SOC | 0.01 |  | -0.05 | 0.07 | .80 |  | 1.11 | 1.00 |
|  | Milder housing damage and higher SOC | 0.01 |  | -0.06 | 0.07 | .96 |  | 1.11 | 1.00 |
|  | Housing destruction and lower SOC | 0.20 |  | 0.06 | 0.34 | < .002 | ** | 1.69 | 1.07 |
|  | Housing destruction and higher SOC | 0.21 |  | 0.06 | 0.37 | .01 | * | 1.72 | 1.00 |
| Incident of fall | No housing damage and higher SOC |  | 1.01 | 0.61 | 1.69 | .96 |  | 1.11 | 1.00 |
|  | Milder housing damage and lower SOC |  | 1.55 | 0.99 | 2.44 | .06 |  | 2.47 | 1.00 |
|  | Milder housing damage and higher SOC |  | 0.95 | 0.57 | 1.57 | .83 |  | 1.29 | 1.00 |
|  | Housing destruction and lower SOC |  | 1.6 | 0.66 | 3.92 | .30 |  | 2.58 | 1.00 |
|  | Housing destruction and higher SOC |  | 1.16 | 0.39 | 3.44 | .79 |  | 1.59 | 1.00 |
| Obesity | No housing damage and higher SOC |  | 0.94 | 0.65 | 1.35 | .73 |  | 1.32 | 1.00 |
|  | Milder housing damage and lower SOC |  | 0.96 | 0.68 | 1.36 | .82 |  | 1.25 | 1.00 |
|  | Milder housing damage and higher SOC |  | 1.18 | 0.84 | 1.67 | .35 |  | 1.64 | 1.00 |
|  | Housing destruction and lower SOC |  | 2.33 | 1.14 | 4.73 | .02 | * | 4.09 | 1.54 |
|  | Housing destruction and higher SOC |  | 1.59 | 0.73 | 3.46 | .24 |  | 2.60 | 1.00 |
| The number of existing diseases | No housing damage and higher SOC | -0.01 |  | -0.12 | 0.09 | .79 |  | 1.11 | 1.00 |
|  | Milder housing damage and lower SOC | 0.11 |  | 0.02 | 0.21 | .02 | * | 1.45 | 1.20 |
|  | Milder housing damage and higher SOC | 0.04 |  | -0.05 | 0.14 | .38 |  | 1.23 | 1.00 |
|  | Housing destruction and lower SOC | 0.01 |  | -0.22 | 0.24 | .93 |  | 1.11 | 1.00 |
|  | Housing destruction and higher SOC | 0.03 |  | -0.21 | 0.26 | .83 |  | 1.20 | 1.00 |
| Mental health |  |  |  |  |  |  |  |  |  |
| Mental distress | No housing damage and higher SOC | -0.43 |  | -0.53 | -0.32 | < .002 | *** | 2.32 | 2.10 |
| (continuos variable of K6 score) | Milder housing damage and lower SOC | 0.16 |  | 0.06 | 0.26 | < .002 | *** | 1.58 | 1.37 |
|  | Milder housing damage and higher SOC | -0.29 |  | -0.39 | -0.19 | < .002 | *** | 1.93 | 1.72 |
|  | Housing destruction and lower SOC | 0.42 |  | 0.19 | 0.66 | < .002 | *** | 2.29 | 1.75 |
|  | Housing destruction and higher SOC | 0.07 |  | -0.16 | 0.29 | .56 |  | 1.33 | 1.00 |
| Depressive symptoms | No housing damage and higher SOC |  | 0.55 | 0.40 | 0.74 | < .002 | *** | 3.04 | 2.04 |
| (>=5 points of GDS-15 score) | Milder housing damage and lower SOC |  | 1.31 | 1.01 | 1.70 | .04 | * | 1.95 | 1.11 |
|  | Milder housing damage and higher SOC |  | 0.7 | 0.53 | 0.93 | .02 | * | 2.21 | 1.36 |
|  | Housing destruction and lower SOC |  | 2.37 | 1.32 | 4.24 | < .01 | ** | 4.17 | 1.97 |
|  | Housing destruction and higher SOC |  | 2.41 | 1.30 | 4.46 | < .01 | ** | 4.25 | 1.92 |
| PTSS (>=6 points of SQD score) | No housing damage and higher SOC |  | 0.52 | 0.32 | 0.85 | < .01 | ** | 3.26 | 1.63 |
|  | Milder housing damage and lower SOC |  | 2.1 | 1.49 | 2.97 | < .002 | *** | 3.62 | 2.34 |
|  | Milder housing damage and higher SOC |  | 1.15 | 0.77 | 1.72 | .48 |  | 1.57 | 1.00 |
|  | Housing destruction and lower SOC |  | 2.95 | 1.56 | 5.56 | < .002 | *** | 5.35 | 2.50 |
|  | Housing destruction and higher SOC |  | 3.78 | 2.00 | 7.17 | < .002 | *** | 7.02 | 3.41 |
| Poor sleep quality | No housing damage and higher SOC | -0.22 |  | -0.32 | -0.11 | < .002 | *** | 1.74 | 1.53 |
|  | Milder housing damage and lower SOC | 0.12 |  | 0.01 | 0.22 | .03 | * | 1.47 | 1.24 |
|  | Milder housing damage and higher SOC | -0.15 |  | -0.25 | -0.04 | < .01 | ** | 1.56 | 1.34 |
|  | Housing destruction and lower SOC | 0.02 |  | -0.21 | 0.25 | .87 |  | 1.16 | 1.00 |
|  | Housing destruction and higher SOC | -0.22 |  | -0.46 | 0.02 | .08 |  | 1.74 | 1.00 |
| Health behavior |  |  |  |  |  |  |  |  |  |
| Less daily walking time | No housing damage and higher SOC | 0.04 |  | -0.06 | 0.14 | .42 |  | 1.23 | 1.00 |
|  | Milder housing damage and lower SOC | 0.07 |  | -0.02 | 0.16 | .14 |  | 1.33 | 1.00 |
|  | Milder housing damage and higher SOC | 0.05 |  | -0.05 | 0.14 | .32 |  | 1.27 | 1.00 |
|  | Housing destruction and lower SOC | -0.01 |  | -0.21 | 0.20 | .95 |  | 1.11 | 1.00 |
|  | Housing destruction and higher SOC | 0.31 |  | 0.09 | 0.53 | < .01 | ** | 1.98 | 1.29 |
| Decreased frequency of going out | No housing damage and higher SOC |  | 0.92 | 0.69 | 1.23 | .56 |  | 1.39 | 1.00 |
| in the past year | Milder housing damage and lower SOC |  | 1.27 | 0.98 | 1.65 | .07 |  | 1.86 | 1.00 |
|  | Milder housing damage and higher SOC |  | 1.03 | 0.78 | 1.36 | .85 |  | 1.21 | 1.00 |
|  | Housing destruction and lower SOC |  | 1.15 | 0.62 | 2.11 | .66 |  | 1.57 | 1.00 |
|  | Housing destruction and higher SOC |  | 1.02 | 0.52 | 1.98 | .96 |  | 1.16 | 1.00 |
| Current smoking | No housing damage and higher SOC |  | 2.86 | 1.39 | 5.85 | < .01 | ** | 5.17 | 2.13 |
|  | Milder housing damage and lower SOC |  | 2.35 | 1.18 | 4.68 | .01 | * | 4.13 | 1.64 |
|  | Milder housing damage and higher SOC |  | 2.87 | 1.42 | 5.78 | < .01 | ** | 5.19 | 2.19 |
|  | Housing destruction and lower SOC |  | 4.91 | 1.20 | 20.11 | .03 | * | 9.29 | 1.69 |
|  | Housing destruction and higher SOC |  | 5.93 | 1.18 | 29.78 | .03 | * | 11.34 | 1.64 |
| Current drinking | No housing damage and higher SOC |  | 1.19 | 0.78 | 1.82 | .42 |  | 1.67 | 1.00 |
|  | Milder housing damage and lower SOC |  | 0.83 | 0.56 | 1.25 | .38 |  | 1.70 | 1.00 |
|  | Milder housing damage and higher SOC |  | 0.91 | 0.61 | 1.37 | .66 |  | 1.43 | 1.00 |
|  | Housing destruction and lower SOC |  | 1.18 | 0.44 | 3.14 | .74 |  | 1.64 | 1.00 |
|  | Housing destruction and higher SOC |  | 1.14 | 0.41 | 3.13 | .80 |  | 1.54 | 1.00 |
| Subjective well-being |  |  |  |  |  |  |  |  |  |
| Low self-reated health | No housing damage and higher SOC | -0.10 |  | -0.20 | 0.00 | .04 | * | 1.42 | 1.16 |
|  | Milder housing damage and lower SOC | 0.11 |  | 0.02 | 0.21 | .02 | * | 1.45 | 1.20 |
|  | Milder housing damage and higher SOC | -0.05 |  | -0.15 | 0.04 | .27 |  | 1.27 | 1.00 |
|  | Housing destruction and lower SOC | -0.01 |  | -0.23 | 0.22 | .95 |  | 1.11 | 1.00 |
|  | Housing destruction and higher SOC | 0.09 |  | -0.13 | 0.30 | .45 |  | 1.39 | 1.00 |
| Unhappiness | No housing damage and higher SOC | -0.36 |  | -0.47 | -0.26 | < .002 | *** | 2.12 | 1.91 |
|  | Milder housing damage and lower SOC | -0.01 |  | -0.11 | 0.09 | .83 |  | 1.11 | 1.00 |
|  | Milder housing damage and higher SOC | -0.33 |  | -0.43 | -0.23 | < .002 | *** | 2.04 | 1.83 |
|  | Housing destruction and lower SOC | 0.28 |  | 0.05 | 0.51 | .02 | * | 1.90 | 1.37 |
|  | Housing destruction and higher SOC | 0.01 |  | -0.23 | 0.23 | .98 |  | 1.11 | 1.00 |
| Cognitive social capital |  |  |  |  |  |  |  |  |  |
| Low expectation for mutual help | No housing damage and higher SOC | -0.11 |  | -0.22 | -0.01 | .03 | * | 1.45 | 1.20 |
| in the local community | Milder housing damage and lower SOC | -0.01 |  | -0.10 | 0.10 | .97 |  | 1.11 | 1.00 |
|  | Milder housing damage and higher SOC | -0.17 |  | -0.27 | -0.07 | < .002 | *** | 1.61 | 1.40 |
|  | Housing destruction and lower SOC | 0.10 |  | -0.13 | 0.32 | .39 |  | 1.42 | 1.00 |
|  | Housing destruction and higher SOC | -0.02 |  | -0.26 | 0.22 | .86 |  | 1.16 | 1.00 |
| Less trust to local residents | No housing damage and higher SOC | -0.01 |  | -0.11 | 0.10 | .95 |  | 1.11 | 1.00 |
|  | Milder housing damage and lower SOC | 0.01 |  | -0.09 | 0.10 | .97 |  | 1.11 | 1.00 |
|  | Milder housing damage and higher SOC | -0.07 |  | -0.17 | 0.03 | .16 |  | 1.33 | 1.00 |
|  | Housing destruction and lower SOC | 0.16 |  | -0.06 | 0.37 | .15 |  | 1.58 | 1.00 |
|  | Housing destruction and higher SOC | 0.10 |  | -0.14 | 0.35 | .41 |  | 1.42 | 1.00 |
| Weak community attachment | No housing damage and higher SOC | -0.10 |  | -0.20 | -0.01 | .04 | * | 1.42 | 1.16 |
|  | Milder housing damage and lower SOC | -0.03 |  | -0.12 | 0.06 | .54 |  | 1.20 | 1.00 |
|  | Milder housing damage and higher SOC | -0.20 |  | -0.30 | -0.11 | < .002 | *** | 1.69 | 1.48 |
|  | Housing destruction and lower SOC | 0.63 |  | 0.41 | 0.84 | < .002 | *** | 2.95 | 2.33 |
|  | Housing destruction and higher SOC | 0.59 |  | 0.35 | 0.83 | < .002 | *** | 2.81 | 2.05 |
| Social well-being |  |  |  |  |  |  |  |  |  |
| Low frequency of meeting friends | No housing damage and higher SOC | -0.04 |  | -0.14 | 0.05 | .39 |  | 1.23 | 1.00 |
|  | Milder housing damage and lower SOC | -0.04 |  | -0.13 | 0.05 | .42 |  | 1.23 | 1.00 |
|  | Milder housing damage and higher SOC | -0.11 |  | -0.20 | -0.02 | .02 | * | 1.45 | 1.20 |
|  | Housing destruction and lower SOC | -0.18 |  | -0.39 | 0.02 | .08 |  | 1.64 | 1.00 |
|  | Housing destruction and higher SOC | -0.24 |  | -0.47 | -0.02 | .04 | * | 1.80 | 1.00 |
| Low frequency of participation | No housing damage and higher SOC | -0.01 |  | -0.09 | 0.08 | .90 |  | 1.11 | 1.00 |
| in sports clubs | Milder housing damage and lower SOC | -0.06 |  | -0.14 | 0.03 | .18 |  | 1.30 | 1.00 |
|  | Milder housing damage and higher SOC | -0.10 |  | -0.18 | -0.01 | .02 | * | 1.11 | 1.00 |
|  | Housing destruction and lower SOC | 0.08 |  | -0.11 | 0.26 | .42 |  | 1.36 | 1.00 |
|  | Housing destruction and higher SOC | -0.03 |  | -0.22 | 0.17 | .78 |  | 1.20 | 1.00 |
| Low frequency of participation | No housing damage and higher SOC | -0.04 |  | -0.13 | 0.05 | .36 |  | 1.23 | 1.00 |
| in hobby clubs | Milder housing damage and lower SOC | -0.04 |  | -0.13 | 0.05 | .35 |  | 1.23 | 1.00 |
|  | Milder housing damage and higher SOC | -0.05 |  | -0.14 | 0.04 | .24 |  | 1.27 | 1.00 |
|  | Housing destruction and lower SOC | 0.10 |  | -0.10 | 0.31 | .32 |  | 1.42 | 1.00 |
|  | Housing destruction and higher SOC | -0.19 |  | -0.42 | 0.03 | .09 |  | 1.66 | 1.00 |
| Small number of friends meeting | No housing damage and higher SOC | 0.08 |  | -0.01 | 0.18 | .08 |  | 1.36 | 1.04 |
| in the past month | Milder housing damage and lower SOC | 0.06 |  | -0.03 | 0.15 | .20 |  | 1.30 | 1.00 |
|  | Milder housing damage and higher SOC | 0.16 |  | 0.07 | 0.26 | < .002 | *** | 1.58 | 1.37 |
|  | Housing destruction and lower SOC | 0.19 |  | -0.01 | 0.40 | .07 |  | 1.66 | 1.00 |
|  | Housing destruction and higher SOC | 0.15 |  | -0.07 | 0.36 | .17 |  | 1.56 | 1.00 |
| Unwillingness to cooperate with | No housing damage and higher SOC | -0.14 |  | -0.24 | -0.03 | .01 | * | 1.53 | 1.31 |
| the local community | Milder housing damage and lower SOC | 0.03 |  | -0.07 | 0.13 | .53 |  | 1.20 | 1.00 |
|  | Milder housing damage and higher SOC | -0.07 |  | -0.18 | 0.03 | .17 |  | 1.33 | 1.00 |
|  | Housing destruction and lower SOC | 0.11 |  | -0.11 | 0.34 | .33 |  | 1.45 | 1.00 |
|  | Housing destruction and higher SOC | 0.09 |  | -0.16 | 0.34 | .48 |  | 1.39 | 1.00 |
| Few emotional social support | No housing damage and higher SOC | -0.01 |  | -0.11 | 0.09 | .87 |  | 1.11 | 1.00 |
|  | Milder housing damage and lower SOC | -0.04 |  | -0.14 | 0.05 | .38 |  | 1.23 | 1.00 |
|  | Milder housing damage and higher SOC | -0.04 |  | -0.13 | 0.06 | .43 |  | 1.23 | 1.00 |
|  | Housing destruction and lower SOC | 0.33 |  | 0.12 | 0.54 | < .01 | ** | 2.04 | 1.51 |
|  | Housing destruction and higher SOC | 0.16 |  | -0.05 | 0.38 | .14 |  | 1.58 | 1.00 |
| Few instrumental social support | No housing damage and higher SOC | 0.06 |  | -0.04 | 0.16 | .25 |  | 1.30 | 1.00 |
|  | Milder housing damage and lower SOC | 0.05 |  | -0.05 | 0.14 | .32 |  | 1.27 | 1.00 |
|  | Milder housing damage and higher SOC | -0.02 |  | -0.12 | 0.08 | .69 |  | 1.16 | 1.00 |
|  | Housing destruction and lower SOC | 0.23 |  | 0.01 | 0.44 | .04 | * | 1.77 | 1.21 |
|  | Housing destruction and higher SOC | 0.08 |  | -0.13 | 0.30 | .45 |  | 1.36 | 1.00 |

Abbreviation: Coef, Coefficient; SOC, Sense of Coherence

* p < .05, ** p < .01, *** p < .002 (threshold after Bonferroni correction for multiple testing: .05/28 outcomes = .002)

Note: Results of multivariate liner or logistic regression models adjusting for loss of loved ones during the disaster, sex, age, educational attainment, working status, equivalized household income, divorced/bereavement, living alone, baseline depressive symptoms (≧ 5p of GDS-15), and baseline outcomes.

Table S5. Two-way interaction effects of housing damage and a continuous variable of sense of coherence on each outcome

|  | Two-way interactions of housing damage and SOC (continuous variable) | | | | | |
| --- | --- | --- | --- | --- | --- | --- |
|  | No housing damage * continuous SOC | | Milder housing damage * continuous SOC | | Housing destruction * continuous SOC | |
| Outcomes | coefficient/odds | | | | | |
| Cognitive health |  |  |  |  |  |  |
| Cognitive decline diagnosed by physicians, coefficient | -0.07 | * | -0.06 |  | 0.04 |  |
| Cognitive decline diagnosed by investigators, coefficient | -0.05 | ** | -0.05 | * | 0.02 |  |
| Physical health |  |  |  |  |  |  |
| Physical disabilities, coefficient | -0.08 | * | -0.06 |  | 0.02 |  |
| Impaired higher-level IADL, coefficient | -0.08 | ** | -0.07 | ** | -0.01 |  |
| Less remained teeth, coefficient | -0.01 |  | -0.01 |  | 0.05 | * |
| Incident of fall, odds | 0.74 | * | 0.77 |  | 0.81 |  |
| Obesity, odds | 1.07 |  | 1.10 |  | 1.29 | * |
| The number of existing diseases, coefficient | -0.05 |  | -0.03 |  | -0.05 |  |
| Mental health |  |  |  |  |  |  |
| Mental distress, coefficient | -0.50 | *** | -0.46 | *** | -0.39 | *** |
| Depressive symptoms, odds | 0.44 | *** | 0.47 | *** | 0.62 | *** |
| PTSS odds | 0.45 | *** | 0.55 | *** | 0.70 | *** |
| Poor sleep quality, coefficient | -0.25 | *** | -0.23 | *** | -0.26 | *** |
| Health behavior |  |  |  |  |  |  |
| Less daily walking time, coefficient | -0.01 |  | 0.01 |  | 0.04 |  |
| Decreased frequency of going out, odds | 0.77 | *** | 0.80 | ** | 0.77 | * |
| Current smoking, odds | 1.26 |  | 1.36 |  | 1.65 | * |
| Current drinking, odds | 0.84 |  | 0.78 | * | 0.84 |  |
| Subjective well-being |  |  |  |  |  |  |
| Low self-rated health, coefficient | -0.19 | *** | -0.17 | *** | -0.18 | *** |
| Unhappiness, coefficient | -0.38 | *** | -0.38 | *** | -0.30 | *** |
| Cognitive social capital |  |  |  |  |  |  |
| Low expectation of mutual help, coefficient | -0.08 | ** | -0.09 | *** | -0.05 |  |
| Less trust to local residents, coefficient | -0.05 |  | -0.06 |  | -0.01 |  |
| Weak community attachment, coefficient | -0.10 | *** | -0.12 | *** | 0.08 | * |
| Social well-being |  |  |  |  |  |  |
| Low frequency of meeting friends, coefficient | -0.01 |  | -0.03 |  | -0.06 |  |
| Low frequency of participation in sports clubs, coefficient | -0.02 |  | -0.04 |  | -0.01 |  |
| Low frequency of participation in hobby clubs, coefficient | -0.04 |  | -0.04 |  | -0.04 |  |
| Small number of friends meeting, coefficient | 0.04 |  | 0.06 | * | 0.07 |  |
| Unwillingness to cooperation, coefficient | -0.11 |  | -0.09 |  | -0.06 |  |
| Few emotional social support, coefficient | -0.05 |  | -0.06 | * | 0.01 |  |
| Few instrumental social support, coefficient | -0.05 |  | -0.06 | * | -0.02 |  |

Abbreviation: Coef, Coefficient; SOC, Sense of Coherence

* p < .05, ** p < .01, *** p < .002 (threshold after Bonferroni correction for multiple testing: .05/28 outcomes = .002)

Note: Results of multivariate liner or logistic regression models adjusting for loss of loved ones during the disaster, sex, age, educational attainment, working status, equivalized household income, divorced/bereavement, living alone, baseline depressive symptoms (≧ 5p of GDS-15), and baseline outcomes.

Table S6. Two-way interaction effects of housing damage (five levels) and a binary variable of sense of coherence on each outcome

|  | Two-way interactions of housing damage (five levels) and SOC (binary variable) | | | | | | | | |
| --- | --- | --- | --- | --- | --- | --- | --- | --- | --- |
|  | No housing damage * higher SOC | Partial * lower SOC | Partial * higher SOC | Minor * lower SOC | Minor* higher SOC | Major * lower SOC | Major * higher SOC | Housing destruction * lower SOC | Housing destruction * higher SOC |
| Outcomes | coefficient/odds | | | | | | | | |
| Cognitive health |  |  |  |  |  |  |  |  |  |
| Cognitive decline diagnosed by physicians, coefficient | 0.02 | 0.03 | 0.02 | 0.38** | -0.03 | 0.25 | 0.21 | 0.57 *** | 0.28 * |
| Cognitive decline diagnosed by investigators, coefficient | -0.01 | 0.02 | -0.02 | 0.19 ** | -0.03 | 0.10 | 0.13 | 0.33 *** | 0.20 * |
| Physical health |  |  |  |  |  |  |  |  |  |
| Physical disabilities, coefficient | -0.03 | 0.07 | -0.02 | 0.30 ** | 0.03 | 0.14 | 0.22 | 0.49 *** | 0.22 |
| Impaired higher-level IADL, coefficient | 0.02 | 0.06 | 0.02 | 0.23 ** | 0.04 | 0.16 | 0.19 | 0.43 *** | 0.20 * |
| Less remained teeth, coefficient | 0.01 | -0.01 | -0.01 | 0.03 | 0.04 | 0.06 | 0.05 | 0.21 ** | 0.24 ** |
| Incident of fall, odds | 0.99 | 1.41 | 0.93 | 1.57 | 1.03 | 2.59 * | 0.51 | 1.61 | 1.10 |
| Obesity, odds | 0.91 | 0.94 | 1.02 | 1.62 | 1.90 * | 0.43 | 1.40 | 2.39 * | 1.60 |
| The number of existing diseases, coefficient | -0.01 | 0.12 * | 0.02 | 0.09 | 0.13 | 0.13 | 0.13 | 0.01 | 0.05 |
| Mental health |  |  |  |  |  |  |  |  |  |
| Mental distress, coefficient | -0.43 *** | 0.14 ** | -0.35 *** | 0.26 * | -0.13 | 0.26 * | 0.07 | 0.45 *** | 0.06 |
| Depressive symptoms, odds | 0.54 *** | 1.18 | 0.66 ** | 2.19 ** | 0.68 | 1.20 | 1.19 | 2.37 ** | 2.67 ** |
| PTSS odds | 0.50 ** | 1.99 *** | 0.82 | 2.64 *** | 2.31 ** | 2.79 ** | 2.39 * | 2.94 *** | 4.01 *** |
| Poor sleep quality, coefficient | -0.22 *** | 0.12 * | -0.15 ** | 0.14 | -0.09 | -0.01 | -0.27 * | 0.01 | -0.23 |
| Health behavior |  |  |  |  |  |  |  |  |  |
| Less daily walking time, coefficient | 0.04 | 0.06 | 0.04 | 0.10 | 0.03 | 0.14 | 0.11 | -0.01 | 0.30 ** |
| Decreased frequency of going out, odds | 0.92 | 1.31 | 1.06 | 0.94 | 0.78 | 1.70 | 1.24 | 1.12 | 0.97 |
| Current smoking, odds | 2.79 ** | 2.25 * | 2.41 * | 2.92 | 4.22 * | 2.02 | 12.27 ** | 4.53 * | 6.83 * |
| Current drinking, odds | 1.21 | 0.88 | 0.89 | 0.56 | 0.84 | 0.86 | 1.84 | 1.22 | 1.16 |
| Subjective well-being |  |  |  |  |  |  |  |  |  |
| Low self-rated health, coefficient | -0.10 * | 0.11 * | -0.03 | 0.19 | -0.18 * | 0.07 | -0.05 | -0.06 | 0.09 |
| Unhappiness, coefficient | -0.37 *** | -0.03 | -0.33 *** | 0.17 | -0.33 *** | -0.11 | -0.29 * | 0.29 ** | 0.01 |
| Cognitive social capital |  |  |  |  |  |  |  |  |  |
| Low expectation of mutual help, coefficient | -0.11 * | -0.02 | -0.16 ** | 0.14 | -0.21 * | -0.10 | -0.12 | 0.11 | -0.01 |
| Less trust to local residents, coefficient | -0.01 | -0.03 | -0.10 | 0.18 | 0.09 | 0.01 | -0.05 | 0.16 | 0.12 |
| Weak community attachment, coefficient | -0.10 * | -0.04 | -0.21 *** | 0.07 | -0.17 * | -0.03 | -0.18 | 0.66 *** | 0.64 *** |
| Social well-being |  |  |  |  |  |  |  |  |  |
| Low frequency of meeting friends, coefficient | -0.04 | -0.05 | -0.13 * | -0.03 | 0.02 | 0.06 | -0.18 | -0.20 | -0.22 * |
| Low frequency of participation in sports clubs, coefficient | -0.01 | -0.04 | -0.13 ** | -0.06 | 0.08 | -0.08 | -0.05 | 0.06 | -0.01 |
| Low frequency of participation in hobby clubs, coefficient | -0.04 | -0.05 | -0.07 | 0.05 | 0.04 | -0.13 | -0.01 | 0.11 | -0.19 |
| Small number of friends meeting, coefficient | 0.09 | 0.07 | 0.18 *** | 0.02 | 0.16 * | -0.08 | 0.03 | 0.22 * | 0.12 |
| Unwillingness to cooperation, coefficient | -0.14 * | 0.03 | -0.08 | -0.07 | -0.19 * | 0.14 | 0.21 | 0.10 | 0.09 |
| Few emotional social support, coefficient | -0.01 | -0.05 | -0.04 | 0.02 | 0.01 | -0.09 | -0.14 | 0.33 * | 0.16 |
| Few instrumental social support, coefficient | 0.06 | 0.04 | -0.03 | 0.08 | 0.06 | 0.04 | -0.13 | 0.24 * | 0.07 |

Abbreviation: Coef, Coefficient; SOC, Sense of Coherence

* p < .05, ** p < .01, *** p < .002 (threshold after Bonferroni correction for multiple testing: .05/28 outcomes = .002)

Note: Results of multivariate liner or logistic regression models adjusting for loss of loved ones during the disaster, sex, age, educational attainment, working status, equivalized household income, divorced/bereavement, living alone, baseline depressive symptoms (≧ 5p of GDS-15), and baseline outcomes.

Table S7. Association between levels of housing damage and residential relocation

|  | No relocation | Relocation | Total |
| --- | --- | --- | --- |
| Housing damage |  |  |  |
| No damage | 1,304 (99.6%) | 5 (0.4%) | 1,309 (100%) |
| Partial | 1,399 (99.6%) | 5 (0.4%) | 1,404 (100%) |
| Minor | 215 (93.9%) | 14 (6.1%) | 229 (100%) |
| Major | 95 (81.2%) | 22(18.8%) | 117 (100%) |
| Destruction | 12 (8.8%) | 124 (91.2%) | 136 (100%) |

Table S8. Definitions of outcomes

| Outcome | Original question and choice | Outcome definition |
| --- | --- | --- |
| **Cogitive health** |  |  |
| Levels of cognitive decline assessed by medical doctors | Under the Japanese long-term care insurance scheme, the applicants requesting long-term care are classified into one of seven levels according to the severity of their cognitive disability. In this scheme, the municipality also asks a panel of physicians to independently assess the cognitive disability level aiming to determine the care requirements of the applicants. | Continuous variable (1 = Suffering some cognitive deficits, but otherwise almost completely independent - 7 = Needs constant treatment in a specialized medical facility) |
| Levels of cognitive decline assessed by trained investigators |  |  |
| **Physical health** |  |  |
| Levels of physical disability | Under the Japanese long-term care insurance scheme, the applicants requesting long-term care are classified into one of eight levels according to the severity of their physical disability. | Continuous variable (1 = Suffering some forms of disabilities, but otherwise almost completely independent - 8 = Spends the whole day in bed and requires assistance to turn over) |
| Impaired higher-level IADL | We used the Tokyo Metropolitan Institute of Gerontology Index of Competence (TMIG-IC), which consists of 13 items that inquired about physical and cognitive performance to assess higher-level instrumental activities of daily living (IADL). We reversed the score on the scale so that higher scores indicate heavy limitations in performing these instrumental activities. | Continuous variable (0 = no functional limitation - 13 = full score of funtional limitation) |
| Less remained teeth | Q. What is the status of your dental health? 1. I have 20 or more natural teeth 2. I have 10 to 19 natural teeth 3. I have 1 to 9 natural teeth 4. I have no natural teeth | Continuous variable |
| Incident of fall | Q. Have you experienced falls in the past year? 1. Many times 2. Once 3. None | Binary variable (0 = none or once, 1 = many times) |
| Obesity | BMI was calculated from self-reported height and weight, and categorized into two groups representing being obese or not according to the World Health Organization classification for Asian populations (≥25.0 BMI) | Binary variable (0 = no obese, 1 = obese) |
| The number of existing diseases | Do you have the following diseases or disabilities? 1. Cancer 2. Heart disease (including arrhythmia) 3. Stroke 4. High blood pressure 5. Diabetes (including a mild form) 6. Obesity 7. Hyperlipidemia 8. Osteoporosis 9. Joint disease / neuralgia 10. Injury / fracture 11. Respiratory disease 12. Gastrointestinal disease 13. Liver disease 14. Mental disease 15. Difficulty swallowing 16. Impaired vision 17. Impaired hearing 18. Elimination problems (including incontinence, frequent urination, difficulty in starting urination, leaking of urine, etc.) 19. Sleep problem | Continuous variable (the total number of diseases) |
| **Mental health** |  |  |
| Mental distress | Mental distress was asked using Kessler Psychological Distress-6. | Continuous variable |
| Depressive symptoms | Depressive symptoms were measured by GDS-15 (Geriatric Depression Scale-15). The score was categorized into lower (four points and under) versus higher (five points and over) risks. | Binary variable (0 = lower risk, 1 = higher risk) |
| Posttraumatic stress symptoms | PTSS was assessed using the Screening Questionnaire for Disaster-Related Mental Health (SQD). This scale is made up of 9 items, with the following predefined cutoff points for PTSS: slightly affected (0–3 points), moderately affected (4–5 points), and severely affected (6–9 points). In the present study, we categorized the response scores into two risk levels (1 = severely affected, 0 = moderately affected and slightly affected). | Binary variable (0 = moderately affected or slightly affected, 1 = severely affected) |
| Poor sleep quality | Q. How do you evaluate your sleep quality over the past month? 1. Very good 2. Good 3. Poor 4. Very poor | Continuous variable |
| **Health behavior** |  |  |
| Less daily walking time | Q. How long do you walk a day on average? 1. Less than 30 minutes 2. 30 to 59 minutes 3. 60 to 89 minutes 4. 90 minutes or more | Continuous variable (reversed score) |
| Decreased frequency of going out in the past year | Q. Has the frequency of your going out decreased since last year? 1. Yes 2. No | Binary variable (reversed score: 0 = no, 1 = decreased) |
| Current smoking | Q. Do you smoke cigarettes? 1. I have never smoked 2. I stopped smoking 5 or more years ago 3. I stopped smoking within the past 4 years 4. I am currently a smoker | Binary variable (0 = I have never smoked, I stopped smoking 5 or more years ago, or I stopped smoking within the past 4 years, 1 = I am currently a smoker) |
| Current drinking | Q. Do you currently drink alcohol? 1. Yes 2. I used to drink 3. No | Binary variable (0 = no or I used to drink, 1 = Yes) |
| **Subjective well-being** |  |  |
| Low self-reated health | Q. How is your current health status? 1. Excellent 2. Good 3. Fair 4. Poor | Continuous variable |
| Subjective unhappiness | Q. How happy are you, with 10 points for extremely happy and 1 point for very unhappy? 10 points Extremely happy, 9, 8, 7, 6, 5, 4, 3, 2, 1 point Very unhappy | Continuous variable (reversed score) |
| **Cognitive socal capital** |  |  |
| Low expectation for mutual help in the local community | Q. Do you think people living in your area try to help others in most situations? 1. Very 2. Moderately 3. Neutral 4. Slightly 5. Not at all | Continuous variable |
| Less trust to local residents | Q. Do you think people living in your area can be trusted in general? 1. Very 2. Moderately 3. Neutral 4. Slightly 5. Not at all | Continuous variable |
| Weak community attachment | Q. How much community attachment do you have to your local community? 1. Very 2. Moderately 3. Neutral 4. Slightly 5. Not at all | Continuous variable |
| **Social well-being** |  |  |
| Low frequency of meeting friends | Q. How often do you see your friends? 1. Almost everyday 2. Two or three times a week 3. Once a week 4. Once or twice a month 5. A few times a year 6. Rarely | Continuous variable |
| Low frequency of participation in sports clubs | Q. How often do you participate in sports clubs? 1. Almost everyday 2. Two or three times a week 3. Once a week 4. Once or twice a month 5. A few times a year 6. Never | Continuous variable |
| Low frequency of participation in hobby clubs | Q. How often do you participate in hobby clubs? 1. Almost everyday 2. Two or three times a week 3. Once a week 4. Once or twice a month 5. A few times a year 6. Never | Continuous variable |
| Small number of friends meeting over the past month | Q. How many friends/acquaintances have you seen over the past month? Count the same person as one, no matter how many times you have seen him/her. 1. None 2. 1 to 2 3. 3 to 5 4. 6 to 9 5. 10 or more | Continuous variable (reversed score) |
| Unwillingness to cooperate with the local community | Q. Do you agree with making it a rule to offer half a day for the interests of the whole area but not for your own interests? 1. I agree 2. Neutral 3. I disagree | Continuous variable |
| Few emotional social support | Q. Do you have someone who listens to your concerns and complaints? 1. Spouse 2. Children living together 3. Children or relatives living apart 4. Neighbor 5. Friend 6. Other | Continuous variable (reversed score: 6 minus the total number of people who listen to the concerns or complaints) |
| Few instrumental social support | Q. Do you have someone who looks after you when you are sick and confined to a bed for a few days? 1. Spouse 2. Children living together 3. Children or relatives living apart 5. Neighbor 6. Friend 6. Other | Continuous variable (reversed score: 6 minus the total number of people who take care of respondents) |
